# Sodium glucose co-transporter 2 inhibitors (SGLT2i) for pediatric kidney disease: the future is near

**DOI:** 10.3389/fped.2025.1521425

**Published:** 2025-01-30

**Authors:** Gilda M. Portalatin, Irene Hong-McAtee, Anna M. Burgner, Edward R. Gould, Tracy E. Hunley

**Affiliations:** ^1^ Division of Nephrology, Vanderbilt University Medical Center, Nashville, TN, United States; ^2^ Division of Pediatric Endocrinology, Vanderbilt University Medical Center, Nashville, TN, United States; ^3^ Monroe Carell Jr. Children’s Hospital at Vanderbilt, Vanderbilt University Medical Center, Nashville, TN, United States; ^4^ Division of Pediatric Nephrology, Vanderbilt University Medical Center, Nashville, TN, United States

**Keywords:** empagliflozin, dapagliflozin, canagliflozin, pediatric chronic kidney disease, SGLT2 inhibitors, chronic kidney disease, IgA nephropathy, proteinuria

## Abstract

The sodium glucose co-transporter 2 (SGLT2) functions in the proximal tubule to reabsorb the bulk of filtered glucose. SGLT2 inhibitors have been developed to promote renal glucose excretion to improve glycemic control in diabetes. Regulatory guidance mandated adequately powered studies to detect increased cardiovascular risk from emerging hypoglycemic medications. This led to recognition of remarkable improvement in cardiovascular and kidney outcomes with SGLT2 inhibition. Moreover, cardiovascular and kidney benefits extend beyond patients with diabetes. The dramatic kidney benefits of SGLT2 inhibitors documented in CKD in adult patients underscores the need for pediatric nephrologists to familiarize themselves with SGLT2 inhibitor therapies. This review explores the currently available body of knowledge regarding the kidney protective effects of SGLT2 inhibitors in adults and mechanisms thought to contribute to improved kidney outcomes. The limited data for SGLT2i treatment in pediatric kidney disease are reviewed and highlight the need for randomized controlled trials of this drug class in pediatric kidney patients as has been done for pediatric diabetes. Dosing patterns for SGLT2 inhibitors from other pediatric settings are reviewed as well as guidance for initiating SGLT2 inhibition in young adults remaining in pediatric nephrology care.

## Introduction/background

The preeminent twentieth century renal physiologist Homer Smith wrote,

“Maintaining the composition of the blood in respect to other constituents devolves largely upon the kidneys. It is no exaggeration to say that the composition of the blood is determined not by what the mouth ingests but by what the kidneys keep;” (1, 2)

Glucose homeostasis is a prime example of Smith's observation. Glucose is freely filtered by the glomerulus into the tubule, and then completely reabsorbed from the ultrafiltrate by the end of the proximal tubule. This is achieved by the sodium glucose co-transporter 2 (SGLT2) in the luminal brush border of the S1 segment which reabsorbs 90% of the filtered glucose. The rest of the glucose is reabsorbed by the sodium glucose transporter 1 (SGLT1) in the final portion of the proximal tubule (S3 segment) (3). The main action of the SGLT2 inhibitors is to enhance renal glucose excretion by inhibiting tubular glucose reabsorption. A crucial advance in the understanding of renal glucose handling was made by Joseph Von Mering in 1885 who showed that phlorizin, a compound that had been isolated from the bark of apple tree roots fifty years prior induced glucosuria and diuresis in dogs and humans (4). It is now understood that phlorizin produced glucosuria through inhibition of renal tubular SGLT2. Though identified before insulin, phlorizin was limited as a diabetes treatment because of its poor oral absorption and its effects to reduce intestinal glucose absorption that causes diarrhea through nonselective inhibition of SGLT1 which is prominently expressed in the gut lumen (5).

By the 1930s, phlorizin's action to block renal glucose reabsorption was localized to the proximal tubule (6, 7). The novel and far-reaching impact of the coupling of glucose transport with sodium transport was first identified in the intestinal brush border and subsequently in the proximal tubule, with sodium facilitating glucose movement up a concentration gradient (8–12). The intestinal Na+ glucose transporter is now recognized to be SGLT1 (13). Antibodies to SGLT1 identified a similar protein in the renal brush border which shared 59% sequence identity with SGLT1 (14, 15). This second renal Na+/glucose cotransporter, termed SGLT2, is located in the proximal tubule S1 segment, is blocked by phlorizin, and has now been shown to be the high-capacity mechanism of renal proximal tubular glucose reabsorption observed a century before (16).

The potential for inhibition of SGLT2 as a novel treatment for diabetes was substantiated in 1999 when the first orally-available SGLT2 inhibitor, T-1095, a synthetic phlorizin O-glycoside analog, lowered blood glucose and hemoglobin A1c in diabetic animal models by increasing urinary glucose excretion (17). Like phlorizin, however, T-1095 also inhibited SGLT1 and led to gastrointestinal disturbances (18). T-1095 and other first generation SGLT inhibitors were abandoned in favor of improved SGLT2 selectivity as well as longer metabolic stability achieved through a C- glycoside structure. One such C-glycoside SGLT2 inhibitor, dapagliflozin was shown to be 30 times more potent for SGLT2 inhibition than phlorizin and effective at once daily dosing (19). Bristol Myers Squibb soon reported SGLT2 inhibition with dapagliflozin as treatment in Type 2 diabetes patients. Improvement in postprandial glucose was noted by day 2 of 14 of dapagliflozin treatment and fasting serum glucose improved significantly by day 13 (20). Longer duration treatment (12 weeks) in patients with Type 2 DM resulted in a decline in hemoglobin A1c of 0.55%–0.9% (21). Healthy subjects administered dapagliflozin showed dose-dependent glucosuria but no decrease in serum glucose reflecting multiple pathways of glucose homeostasis (22).

In 2012, the European Medicines Agency (EMA) approved dapagliflozin for use in adults with type 2 diabetes, both as monotherapy and in combination with other anti-diabetic medications. In 2014, the US Food and Drug Administration (FDA) followed suit as well as approving another SGLT2 inhibitor, empagliflozin. Over the next couple of years, multiple SGLT2 inhibitors were approved for unaccompanied treatment of type 2 diabetes (canagliflozin and empagliflozin) as well as in combination with metformin. In 2018, Merck was approved for unaccompanied use of the SGLT2 inhibitor ertugliflozin as well as combined with metformin and also in combination with the DPP-4 inhibitor sitagliptin (23). In Jan 2023 bexagliflozin was approved for Type 2 DM treatment. Importantly, the first recognition of SGLT2 inhibition benefits beyond diabetes control was by the Empagliflozin Cardiovascular Outcome Event Trial in Type 2 Diabetes Mellitus Patients (EMPA-REG OUTCOME). The study reported that the addition of empagliflozin decreased cardiovascular death by 38% and all-cause mortality by 32%, compared to placebo (24).

By exploring the improved kidney outcomes with SGLT2 inhibition in adults and the limited data available for SGLT2 inhibition in children and youth with kidney disease, this review aims to highlight the need for robust and well-funded evaluation of SGLT2 inhibitors for treatment of kidney disease in pediatric patients. Moreover, the review aims to facilitate the use of SGLT2 inhibitors for young adults remaining in pediatric nephrology care, for whom SGLT2 inhibitors are presently available.

## Efficacy in chronic kidney disease

Clinical trials indicate that SGLT2 inhibitors in adults have wide-ranging cardiovascular and kidney effects beyond their glucose lowering action. These benefits are also observed in individuals without diabetes mellitus. These effects were recognized due to the mandatory cardiovascular outcome trials required by the United States Food and Drug Administration and the European Medicines Agency for all new antidiabetic drugs which required inclusion of high-risk groups including those with renal impairment (25). The EMPA-REG OUTCOME trial published in 2015 showed that adults with type 2 diabetes and cardiovascular disease treated with empagliflozin had a lower rate of cardiovascular death, all-cause mortality, and hospitalization for heart failure when compared to placebo (24). Two years later, the CANVAS trial demonstrated that in patients with type 2 diabetes who were 30 years of age or older with a history of symptomatic cardiovascular disease or patients 50 years of age or older with at least two risk factors for cardiovascular disease, treatment with canagliflozin led to a decrease in the rate of the composite of cardiovascular death, nonfatal myocardial infarction, or nonfatal stroke (26). Notably, the CANVAS trial also found less progression of albuminuria and a decreased risk of the composite outcome of 40% reduction in eGFR, need for renal replacement therapy, or death from renal causes in persons who received canagliflozin, suggesting a role for SGLT2 inhibitors in kidney protection. Although the DECLARE-TIMI trial of dapagliflozin in patients 40 years of age or older with type 2 diabetes at risk for cardiovascular disease or established cardiovascular disease found no difference in the risk of major adverse cardiovascular events (MACE) compared to placebo, it found a decreased risk of heart failure hospitalization (27). Moreover, DECLARE-TIMI reported kidney protection with SGLT2 inhibitors in secondary analysis with the risk halved for GFR drop of 40% to <60 ml/min/1.73 m^2^ or ESRD or death from renal causes in dapagliflozin treated Type 2 diabetics.

Remarkably, the kidney protective effects were observed even in individuals without diabetes. The first of these observations came in the CREDENCE trial. Indeed, this study was terminated early due to efficacy. It was conducted in type 2 diabetes patients > 30 years of age, with an eGFR of 30-<90 ml per minute per 1.73 m^2^ (CKD), a urinary albumin to creatinine ratio of >300–5,000 mg/g (macroalbuminuria), and already treated with renin-angiotensin system blockade. Canagliflozin-treated patients had a 30% decrease in relative risk of the composite outcome of end-stage kidney disease, doubling of serum creatinine, or death from renal or cardiovascular outcomes if treated with canagliflozin in just median 2.6 years follow up (28). Another trial, DAPA-CKD enrolled patients 18 years of age or older with an eGFR 25–75, a urine albumin to creatinine ration between 200 and 5,000 mg/g, and who were stable on maximum tolerated ACE inhibitor or ARB with type 2 diabetes (two thirds of patients) or without diabetes. Patients were randomized to dapagliflozin or placebo. Irrespective of the patient's type 2 diabetes status, dapagliflozin treatment led to a significantly decreased risk of the composite of decrease in eGFR of 50%, end-stage kidney disease, or death from renal or cardiovascular causes with hazard ratio 0.61 (95% CI: 0.51–0.72; *P* < 0.002) (29).

EMPA-KIDNEY enrolled patients 18 years of age or older with or without diabetes (of any type) who were randomized to empagliflozin or placebo. Patients with an eGFR between 20 and <45 were enrolled irrespective of albuminuria and patients with an eGFR between 45 and <90 who had a urine albumin to creatinine ratio of at least 200 mg/g. Treatment with empagliflozin led to a decreased risk of progression of kidney disease or death from cardiovascular causes with results consistent across eGFR subgroups and irrespective of diabetes diagnosis. Benefit was most pronounced in proteinuric patients with albumin to creatinine ration above 300 mg/gm (30). Notably, 1,669 patients in EMPA-KIDNEY had glomerular disease as the cause of their CKD: 817 IgA nephropathy, 195 FSGS, and 657 had other causes of glomerulonephritis. The primary outcome of kidney disease progression or cardiovascular death was decreased similarly across all main categories of cause of kidney disease (31). In addition, empagliflozin treatment for two years led to a ∼50% reduction in decline in GFR without difference between primary kidney disease groups (diabetes, hypertension/renovascular, glomerular, and other/unknown).

While most of the published trials report better kidney outcomes with SGLT2i, this is not universal. The much smaller and shorter DIAMOND study was conducted in patients without diabetes and mean GFR 55–60 ml/min/1.73 m^2^ with dapagliflozin for 6 weeks (*N* = 53). No change in proteinuria was observed and dapagliflozin treatment decreased GFR by 6.6 ml/min/1.73 m^2^ which was reversed upon discontinuation of medication (32).

Reduction of proteinuria with SGLT2 inhibition has been observed repeatedly in diabetic kidney disease and likely contributes to the improved renal outcomes (33). In the CANVAS trials involving over 10,000 type 2 diabetic patients, progression of albuminuria was assessed as 30% increase in albuminuria or increase from normoalbuminuria to micro- or macroalbuminuria or microalbuminuria to macroalbuminuria. Among patients treated with canagliflozin for 3–4 years, remarkable improvement in proteinuria was observed: progression of albuminuria was decreased (hazards ratio 0.73; 95% CI: 0.67–0.79) (26). Conversely, regression of albuminuria (using the reverse definitions) was more common in canagliflozin-treated patients (Hazards ratio 1.7 (95% CI: 1.51–1.91). In the CREDENCE trial of canagliflozin in type 2 DM patients with macroalbuminuria and decreased GFR (30–90 ml/min/1.73 m^2^), urine albumin to creatinine ratio was decreased by 31% in patients receiving canagliflozin (28). In the DELIGHT trial in patients with type 2 DM and albuminuria (30–3,500 mg/gm creatinine), patients treated with dapagliflozin for six months (*n* = 140) showed a 21% reduction in albuminuria vs. placebo-treated patients (34). The EMPA-Kidney trial, in whom patients had less albuminuria overall, with median albumin to creatinine ration 329 mg/gm, showed a 19% decrease in albuminuria in empagliflozin-treated patients (30). Even among diabetics with nephrotic range proteinuria (urine albumin to creatinine ratio ≥ 2,200 mg/gm), treatment with empagliflozin resulted in a hazard ratio of 2.30 (95% CI: 1.34–3.93) for at least a 30% decrease in proteinuria compared to placebo (35). Moreover, median predicted time to ESKD was increased from 5 to 10 years in the empagliflozin-treated.

Data for SGLT2 inhibitor reduction in non-diabetic kidney disease is less plentiful but emerging. Among Spanish adults with biopsy-proven glomerular disease (*n* = 493), SGLT2 inhibition resulted in a mean 48% proteinuria reduction at 1 year follow up, occurring less often in those with serum albumin < 3.5 g/L. As in diabetic disease those with ≥30% proteinuria reduction showed slower decline in GFR (36). Among Egyptian adults with varied glomerulonephritis (*N* = 25), treatment with empagliflozin decreased progression of proteinuria (Odds ratio 0.65; 95% CI: 0.55–0.72) (37).

## Mechanisms of SGLT2 inhibitors in the kidney

Multiple studies including CREDENCE, DAPA-CKD, and EMPA-Kidney have demonstrated beneficial effects of SGLT2-inhibitors: lower risk of CKD progression, reduction of proteinuria, and lower risk of cardiovascular mortality. However, one must question, “How does this all happen?” Sodium glucose transporters located on the luminal side of the proximal tubule are responsible for nearly all glucose reabsorption. Diabetes upregulates the SGLT co-transporters, increasing glucose reabsorption.

### Augmented tubuloglomerular feedback

**Figure 1:**
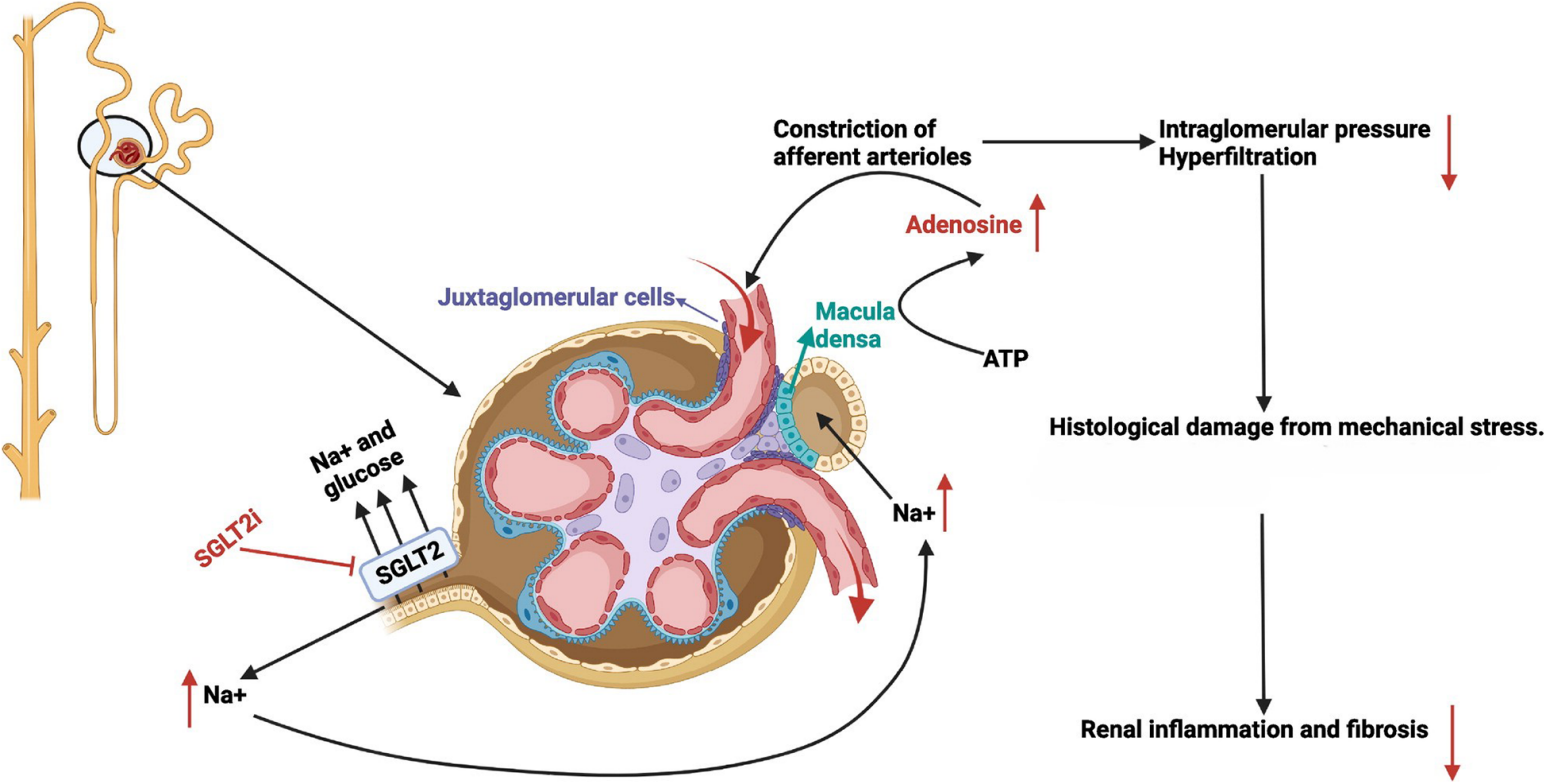
Mechanism of action of sodium glucose co-transporter 2 inhibitors through enhanced tubular glomerular feedback. With permission from Ref. (40, p. C664). A nephron is shown on the left with the glomerulus, juxtaglomerular apparatus, and early proximal tubule enlarged on the right. SGLT2 inhibitors (red text) block the reabsorption of Na+ and glucose in the proximal tubule leading to glucosuria and increased Na+ delivery to the distal tubule and macula densa. As a result, macula densa cells release ATP which is hydrolyzed locally to adenosine (red text). Adenosine acts on adjacent smooth muscle adenosine receptors to constrict the afferent arterioles. Decreased afferent arteriolar blood flow lowers intraglomerular pressure and decreases glomerular filtration, thus ameliorating proteinuria, podocyte derangement and loss, as well as downstream inflammation and renal fibrosis.

An important mechanism for the kidney protective effect SGTL2-inhibitors involves tubuloglomerular feedback (TGF) whereby tubular flow and composition modulate arteriolar vascular tone and thus the glomerular filtration. Under normal conditions, TGF acts to limit glomerular filtration when flow in the distal tubule is ample. Diabetes blunts tubuloglomerular feedback. Prevailing hyperglycemia upregulates proximal tubular sodium-glucose cotransporter and increases glucose, sodium, and chloride reabsorption. The ensuing reduction in sodium and chloride delivery to the distal nephron and macula densa results in decreased afferent arteriolar tone (vasodilatation) and increased glomerular perfusion and filtration (38). Support for this scenario is provided by studies in spontaneously diabetic mice (Ins2^+/Akita^) that show single nephron GFR is two-fold higher than non-diabetic controls, (15.8 ± 6.8 nl/min vs. 4.9 ± 1.3 nl/min, control). Importantly, treatment with the SGLT2 inhibitor empagliflozin normalized the increased GFR in diabetic mice (8.0 ± 3.3 nl/min) (39). The underlying mechanism involves local vasoactive adenosine which acts on smooth muscle cells to induce vasoconstriction of the afferent arteriole, resulting in decreased glomerular blood flow (Figure 1). In another preclinical diabetes model, *in vivo* imaging was able to visualize afferent arteriolar vasoconstriction after SGLT2 inhibitor treatment and decrease in single nephron GFR (41). Moreover, SGLT2 inhibitors can act synergistically to reduce intraglomerular pressure and hyperfiltration by the concomitant efferent vasodilation by renin-angiotensin-aldosterone blockade (42).

Even in the absence of diabetes, SGLT2 inhibition offers kidney protection at least in part through tubulo-glomerular feedback. In the 5/6 nephrectomy rat model of chronic kidney disease in which diabetes does not feature, SGLT2 inhibition with empagliflozin ameliorated glomerular hypertrophy, glomerulosclerosis and interstitial fibrosis as effectively as inhibition of the renin angiotensin system with telmisartan (43). Moreover, preservation of creatinine clearance over time correlated directly with higher urinary adenosine levels (indicating improved TGF and afferent arteriolar vasoconstriction). Higher adenosine levels also correlated with minimization of interstitial fibrosis pointing to overall renal preservation.

### Renal tubule effects

Proximal tubular cells are the exclusive site of glucose reabsorption from the glomerular ultrafiltrate. They are, therefore, dramatically impacted by persistent hyperglycemia on a cellular level. Histologically, the tubulointerstitial changes include proximal tubular cell basement membrane thickening, hyperplasia, and hypertrophy, eventually resulting in atrophy as diabetic disease progresses. Direct effects of high glucose levels result in activation of inflammatory and profibrotic effects in proximal tubular cells (PTCs). Thus, PTCs cultured in a high glucose environment showed increased expression of Toll-like receptor 4, increased nuclear DNA binding of transcription factors NF-κB and AP-1 as well as increased expression of collagen IV and secretion of IL-6—all of which were attenuated by SGLT2 inhibition with empagliflozin (44). The damaging effects on proximal tubular cell growth and function have been linked to cytokine TGFβ, resulting from increased glucose exposure and intracellular influx (45). SGLT2 inhibitors moderate this destruction via blockade of glucose transporters (46). SGLT2 inhibitors may also mitigate transition from renal monocytes to inflammatory M1 macrophages which contribute to renal interstitial fibrosis (40). Among patients with diabetes inadequately controlled by metformin, SGLT2 inhibition reduced circulating biomarkers of inflammation and fibrosis compared to treatment with the insulin secretagogue glimepiride in 1–2 years follow up. Thus, plasma samples from canagliflozin treated patients showed lower levels of circulating TNF Receptor 1, IL-6, Matrix metalloproteinase 7, and fibronectin 1 despite identical hemoglobin A1c levels to glimepiride treated patients (47).

Kidney biopsies from teens and young adults with type 2 diabetes and healthy controls were interrogated with single cell transcriptional profiling (48). The renal biopsies were not performed for clinical reasons. Among patients with diabetes, some had been treated with SGLT2 inhibition allowing comparison to SGLT2i-untreated patients. As expected, a variety of transcripts along the renal tubule were differentially regulated by diabetes and SGLT2 inhibition identifying significant alterations in the mTORC1 pathway. Phosphorylation of ribosomal protein S6 (rpS6), a marker of mTORC1 signaling activation was detected in the proximal and distal tubules of the diabetic patients, indicating mTORC1 activation in SGLT2i untreated diabetics. Phosphorylated rpS6 staining was normalized in those treated with SGLT2 inhibitors. Thus, even before overt kidney disease is present, renal tubular cells in Type 2 DM show upregulation of mTORc1 that can be ameliorated by SGLT2 inhibition. As mTORc1 functions at a nexus of metabolic, growth, immune and fibrogenic signaling, the implications of its treatment extend beyond diabetic kidney disease with potential relevance for all progression of chronic kidney disease (49).

### Impact on mesangial cells

Increased extracellular glucose concentration results in dysfunction of other renal cell lines. Mesangial cells are specialized pericytes which have a role in regulation of glomeruli circulation that ensures maintenance of constant blood flow. Diabetic nephropathy is marked by progressive mesangial expansion, nodular accumulation of mesangial matrix (Kimmelstiel Wilson nodules) and resultant capillary microaneurysms (50). Using *in vitro* studies, mesangial cells from diabetic rats showed loss of contractile response to angiotensin II, cell swelling, over-production of extracellular matrix, and apoptosis. The mesangial dysfunction resulted in induction of glomerular hyperfiltration and development of glomerular microaneurysms. Normalization of these effects was described with SGLT2 inhibitors indicating renoprotective effects on mesangial cells (51). Mesangial cells cultured with high glucose showed increased TGF beta and fibronectin that was normalized by treatment with canagliflozin (52). Interestingly, aside from mesangial cells, SGLT2 is also expressed in retinal pericytes that maybe involved in diabetic microaneurysm formation (51). It is therefore notable that the incidence of sight threatening retinopathy was significantly decreased in type 2 diabetic patients treated with SGLT2 inhibition compared to DPP4 inhibition, pioglitazone or sulfonylureas (53).

### Impact on podocyte function

Podocytes are critical in maintaining the glomerular filtration barrier and proteinuria indicates their functional decline, common in all chronic kidney disease. In addition to improved glucose control and decreased blood pressure, SGLT2 inhibition significantly decreased podocyte shedding into the urine which was not seen in control treated patients (54). Mice with proteinuria induced by protein overload showed increased glomerular staining for SGLT2 which colocalized with nephrin confirming its podocyte location. Animals treated with dapagliflozin showed significant preservation of glomerular structure and amelioration of proteinuria to the same degree as ACEi treated animals (55).

Upregulation of SGLT2 expression in cultured human podocytes resulted in observed marked remodeling of the cytoskeleton caused by NF-κB activation. SGLT2 inhibition with dapagliflozin ameliorated cytoskeleton rearrangement and improved overall podocyte dysfunction (55). Ongoing research indicates that human and wild type mouse podocytes in culture express SGLT2. Podocyte damage in animal models of non-diabetic disease such as the Col4a3 knockout model of Alport Syndrome prone to lipotoxicity showed that empagliflozin was protective of lipotoxicity-induced podocyte apoptosis (56). Similar benefits were reported in podocytes from lupus prone mice and patients with lupus nephritis (57). Some of the impact of SGLT2 inhibition to various non-diabetic kidney disease may relate to the degree of SGLT2 expression in podocytes in various clinical settings. In one pilot trial of SGLT2 inhibition in FSGS, proteinuria did not decrease after eight weeks of treatment with dapagliflozin which the authors theorized might relate to podocyte downregulation of SGLT2 mRNA which they observed in a archival renal biopsies of FSGS (58).

### Role in inflammation

The benefits of SGLT2 inhibition may reflect alterations in immune function (59). Reduction in IL-6 levels has been repeatedly observed in clinical trials of SGLT2 inhibitors. Given the importance of IL-6 to vascular pathophysiology, IL-6 reduction likely contributes to the cardiovascular and renal benefits of SGLT2 inhibition (60). In lupus prone MRL/*lpr* mice treated with empagliflozin for 20 weeks, dramatic improvement in serum creatinine, proteinuria, and renal histology was observed compared to untreated animals (57). Unexpectedly, empagliflozin treated mice also showed significantly lower IgG levels and anti dsDNA levels. In another study, some complement components were altered by SGLT2 inhibition. Thus, in rats with 5/6 nephrectomy the upregulation of C1q subunits A and C were normalized during treatment with empagliflozin and was thought to contribute to renoprotection (43). Canagliflozin was recently shown to impair T cell function through diminished T cell receptor signaling, reduced activation, reduced proliferation, altered metabolism and decreased cytokine production (61). Dapagliflozin did not show these same effects and SGLT2 expression in T cells is not robust or absent, so Canagliflozin's effects in T cells is likely off-target, though not yet clarified. Calls have been made for the repurposing of canagliflozin for autoimmune diseases (62).

## SGLT2 inhibition in specific kidney diseases

The large data sets available from CKD treatment trials with SGLT2i allow subsequent analysis of differential impact in various primary renal diseases. Together with trials conducted in more restricted groups of kidney diseases, assessment of the potential for benefit for SGLT2i in specific clinical entities is rapidly emerging.

### IgA nephropathy

Because it is the most common chronic primary glomerular disease, IgA nephropathy (IgAN) contributes significantly to prevalent CKD (63). Therefore, available SGLT2i treatment data for IgAN is sufficient to draw conclusions regarding efficacy. The DAPA CKD trial enrolled 270 patients with IgAN who fulfilled its inclusion criteria of with GFR 25–75, urine albumin to creatinine ration 200–5,000 mg/g, stable dose of ACEI/ARB and no immunotherapy for 6 months before enrollment. They were randomized to dapagliflozin 10 mg daily or placebo. Dapagliflozin dramatically reduced (by ∼75%) those patients reaching the composite renal endpoint of ESKD, 50% decrease in GFR or kidney disease-related death (Hazard ratio 0.24 [0.09, 0.65], *P* = 0.002 (64). Secondary analysis of the EMPA-KIDNEY trial included 870 IgAN patients with GFR 45–90, UAC >200 mg/g, with use of single ACEI/ARB required for all participants. Those taking prednisone ≥45 mg daily or IV immunosuppression in the preceding 3 months were excluded. Kidney disease progression (development of ESKD or GFR < 10 or 40% decrease in GFR) was again dramatically reduced in those IgAN patients receiving empagliflozin [Hazard ratio 0.67 (95% CI: 0.46–0.97)]. The decline in GFR over time was lessened by 30%–40% among the empagliflozin treated IgAN patients (31). Recently, a large observational European cohort of glomerulonephritis patients was published of whom all were on some dose of ACEI/ARB and all received SGLT2 inhibitors. The study included 203 patients with IgAN or IgAV who showed a 50% decrease in proteinuria after 12 months of SGLT2i treatment (36). This is a notable finding given that proteinuria reduction vs. persistence is a satisfactory surrogate marker for outcomes in IgAN (65). A smaller Chinese study shows that even in patients on maximal doses of ACEI/ARB, further proteinuria reduction (∼25%) was seen in a short duration of SGLT2i treatment (6 months) (66). Thus, multiple sources of evidence align to indicate that SGLT2 inhibition greatly benefits patients with IgAN by reducing proteinuria and slowing decline in kidney function.

### FSGS

FSGS is a glomerular disease which is often resistant to treatment and continues to constitute a common diagnosis leading to ESKD, especially in pediatric patients. Data from SGLT2i treatment of FSGS continue to accrue from adult studies. In an eight-week pilot trial of dapagliflozin in ten adult FSGS patients, all treated with ACEi/ARB, no decrease in proteinuria was observed (58). A prespecified analysis of the DAPA-CKD trial showed a promising Hazard ratio of 0.67 for biopsy-proven FSGS patients (*N* = 104) with regard to reaching ESKD or experiencing ≥40% drop in GFR, the kidney composite outcome. Unfortunately, the 95% confidence interval for the HR was 0.19–2.44 and so intersected the HR 1.0/Forest plot Line of no effect. The authors hypothesized that small sample size was obscuring dapagliflozin benefits in FSGS, but disease heterogeneity and/or low/no efficacy may have contributed as well (67). Among EMPA-KIDNEY patients with FSGS and a larger sample size (*N* = 195), empagliflozin did not appear to decrease the kidney progression outcome (ESKD or ≥40% GFR decrease). In fact, the Hazard ratio 1.35 (0.65–2.81) favored placebo over empagliflozin. Analyses of GFR slope for FSGS patients in EMPA-KIDNEY did appear to favor empagliflozin slightly over placebo, but in all subgroup analyses of FSGS, the 95% CI intersected the Forest plot line of no effect tempering enthusiasm for empagliflozin in FSGS (31). More promising results are seen in the large observational European cohort of glomerulonephritis patients described above, of whom all were on ACEI/ARB and all received SGLT2 inhibitors. 56% of primary FSGS patients (*N* = 32) and 83% of secondary FSGS (*N* = 58) showed a ≥30% reduction in proteinuria (36). The optimism for SLGT2 inhibitor treatment generally must remain cautious for FSGS treatment given currently available evidence, though disease heterogeneity of FSGS raises the possibility that some patients may benefit from SGLT2 inhibition.

### Obesity related glomerulopathy (ORG)

ORG is a chronic kidney disease characterized by glomerular hyperfiltration and proteinuria which occurs in obese individuals (68). The incidence of ORG has increased at least 10-fold in the last several decades and occurs in pediatric patients as well (69, 70). Histologically, ORG is characterized by glomerulomegaly and podocyte hypertrophy. The glomerular enlargement effects the increase in GFR. Given that podocytes are terminally differentiated and do not proliferate, their number does not increase and their density declines in the enlarging glomerulus. Podocytes undergo increasing stress, fail and detach, leading to glomerulosclerosis.

Multiple components of the pathophysiology of ORG may be amenable to treatment with SGLT2i (71). The glomerular hyperfiltration of severe obesity entails prominent afferent arteriolar vasodilation, and as above, SGLT2 inhibition curbs afferent arteriolar vasodilation through natriuresis and increased tubuloglomerular feedback (72). Alterations in adipokines in obesity also contribute to renal damage. Leptin stimulates TGF beta overexpression. Resistin upregulates inflammatory cytokines such as TNF alpha, Il-6 and IL-12 contributing to low grade chronic inflammation. By contrast, adipose-derived adiponectin declines with worsening obesity, thus decreasing its normal renoprotection to injured podocytes (73). Dapagliflozin, empagliflozin, and canagliflozin have been shown to ameliorate these metabolic derangements, increasing circulating levels of adiponectin and decreasing leptin, TNF- alpha and IL-6 (74). Given that obesity worsens outcomes of other glomerular disease beyond ORG, it is noteworthy that a recent trial of SGLT2 inhibition in glomerulonephritis patients showed higher proteinuria reduction with SGLT2 inhibition in patients with increased BMI (36, 75).

### Alport syndrome

Alport syndrome, or hereditary nephritis, is a chronic kidney disease frequently encountered in pediatric nephrology in which the glomerular basement membrane is impaired by mutations in the collagen IV genes COL4A3, COL4A4, and COL4A5 which also impair collagen containing structures in the ear and eye. Beginning with microscopic hematuria, AS progresses to albuminuria, overt proteinuria and then declining kidney function (76). Eighty percent of AS is caused by mutations in COL4A5 located on the X chromosome leading to worse outcomes in males. Fifty percent of males reach ESRD by age 25 whereas 40% of females reach ESRD by age 80 (77, 78). Treatment with ACEi reduces proteinuria and delays time to kidney failure and more so the higher the GFR at ACEi initiation, making timely nephroprotection imperative (79).

Potential benefit of SGLT2 inhibition in AS has been proposed, utilizing SGLT2i-induced afferent arteriolar vasoconstriction to lessen glomerular pressure exerted through the diseased glomerular basement membrane (80). Very recently, results were published from an international observational study in Alport patients of SGLT2 inhibitor therapy added on to renin angiotensin inhibition in 112 AS patients, aged 38 ± 14 years (81). Allowing for the limited duration of the study, reduction in proteinuria is taken as a surrogate marker for reduced risk of progression. Baseline albuminuria of 1,797 ± 1,600 mg/g creatinine decreased to 1,197 ± 978 mg/g creatinine after 1–3 months of treatment (*P* = 0.002) and remained at essentially the same level in those followed to 4–8 months and 9–15 months. In patients followed for two years, the baseline albuminuria of 2,127 mg/g creatinine dropped to 1,903 ± 1,371 mg/g creatinine and was not significant, possibly due to low patient number (*N* = 7). Subgroup analysis of the ten pediatric patients (age 15 ± 3 years) showed stable blood pressure after 4 ± 5 months of SGLT2i therapy (BP 116 ± 13/72 ± 11 to 117 ± 13/71 ± 13 mm Hg). GFR decreased from 119 ± 32 ml/min/1.73 m^2^ to 107 ± 36 ml/min/1.73 m^2^. In the two patients with overt proteinuria, urine albuminuria decreased from 1,426 ± 1,247 mg/g creatinine to 641 ± 190.

A multicenter, randomized, double-blind, placebo-controlled trial of dapagliflozin in AS has recently been announced by the German Society of Pediatric Nephrology. The DOUBLE PRO-TECT Alport Trial will enroll pediatric patients (age 10–17 years) and young adults (18–39 years) who despite being on stable maximum dose RAS inhibitor have persistent albuminuria (>300 mg/g creatinine for children > 500 mg/g creatinine for adults). Importantly and differing from previous clinical trials of SGLT2 inhibition, DOUBLE PRO-TECT will enroll patients with mild CKD (Stage 1–2) but at high risk of CKD progression. Treatment to placebo randomization will be 2:1 and treatment will last 48 weeks with reevaluation 4 weeks after discontinuation. Open-label SGLT2i continuation after that will be offered in adults (82).

### Lupus nephritis

Lupus nephritis patients were excluded from the large trials of SGLT2 inhibitors in chronic kidney disease (28, 29). Available data of SGLT2i use in Lupus has been sparse but is promising. Five adult SLE patients with eGFR 34–94 ml/min/1.73 m^2^ experienced a 50% reduction in proteinuria after eight weeks treatment with empagliflozin 10 mg (83). Similarly, among nine adult patients with eGFR 60–126.8 ml/min/1.73 m^2^, two months of SGLT2 inhibitor treatment resulted in significant decreases in proteinuria ranging from 29.6–96.3% (57). Database assessment of the U.S. Collaborative Network of the TriNetX clinical data platform identified *N* = 3,550 patients with SLE and type 2 DM. Those treated with SGLT2 inhibitors had significant reduced risk for lupus nephritis [adjusted hazard ratio (AHR) 0.55; 95% CI: 0.40–0.77], dialysis (AHR 029; 95% CI: 0.17–0.48), kidney transplant (AHR 0.14, 95% CI: 0.03–0.62), heart failure (AHR 0.65, 95% CI: 0.53–0.78) and all-cause mortality (AHR 0.35, 95% CI: 0.26–0.47) (84).

### Autosomal dominant polycystic kidney disease (ADPKD)

Patients with ADPKD were also excluded from the large trials of SGLT2 inhibitors in chronic kidney disease (29, 30). SGLT2i effects of natriuresis and diuresis would be thought to lead to increased circulating vasopressin. Importantly, antagonism of vasopressin effect on the kidney by tolvaptan slows cyst growth, preserves kidney function and is FDA approved for kidney preservation in progressive ADPKD (85). Indeed, preclinical studies show an increase in urinary vasopressin during SGLT2i treatment (86). Nonetheless, the pleiotropic benefits of SGLT2 inhibitors could be hypothesized to outweigh the negative impact of vasopressin increase. Further consideration of SGLT2i kidney preservation in ADPKD has been advocated (87). Two recent studies with dapagliflozin in ADPKD help address these issues (88, 89). Despite small numbers (*N* = 7 and *N* = 10, respectively) and short duration (102 ± 20 days and 20 months), both studies showed a significant increase in height adjusted total kidney volume during treatment, the most useful surrogate marker for kidney progression in ADPKD (90). Indeed, the latter study was able to demonstrate an increase in the rate of kidney growth during dapagliflozin treatment. These preliminary studies do not support the use of SGLT2 inhibition for kidney preservation in ADPKD at this time and may even sway clinicians to choose other hypoglycemic treatments in those patients with diabetes and ADPKD.

## SGLT2 inhibitor use in the young

The dramatic kidney benefits of SGLT2 inhibitors documented in CKD in adult patients underscores the need for pediatric nephrologists to familiarize themselves with SGLT2 inhibitor therapies. SGLT2 inhibitors are EMA/FDA approved for use in adult CKD and are, therefore, available for patients older than 18 still under pediatric nephrology care. While health-care transition from pediatric to adult nephrology is a widespread goal, the reality of formal transition care may remain aspirational in many centers (91). The development of the “CKiD under 25” GFR estimating equations and calculator for children through to young adults attests to the reality that patients with CKD above the age of 18 may still be cared for by pediatric nephrologists (92). As such, young adults still under pediatric nephrology care require access to all therapeutic options to improve kidney outcomes, including SGLT2 inhibitors.

### Pediatric diabetes

The long-term harm from type 2 diabetes in the pediatric population is magnified, because the younger a child is at diagnosis with T2DM, the higher incidence of diabetes complications even in young adulthood (93). While SGLT2 inhibitors have become widely used in adults, until 2019 with the advent of glucagon-like peptide-1 (GLP-1) receptor agonists such as liraglutide, the only approved treatments for pediatric patients/children with type 2 diabetes mellitus (T2DM) were insulin and metformin. GLP-1 receptor agonists, however, have several obstacles to their use in pediatric patients. They are injectable, cost-prohibitive, and often in short supply, in part because they are also used in the treatment of obesity. SGLT2 inhibition is now an option for T2DM treatment in pediatric patients. On June 20, 2023, the oral SGLT2 inhibitor empaglifozin was FDA approved for pediatric use down to the age of 10 years (94). Empagliflozin was shown to be safe and efficacious in the DINAMO study, a double-blind, placebo-controlled trial performed in 108 sites in 15 countries in 262 youths aged 10–17 years with type 2 diabetes mellitus (95). Fifty-two pediatric patients were treated with empaglifozin at doses of 10 and 25 mg and after 26 weeks showed a significant and clinically meaningful reduction in hemoglobin A1c not seen for linagliptin. Thus, mean decrease in HgbA1c was 0.84% for empagliflozin vs. placebo (range −1.50–−0.19; *P* = 0.012) whereas the linagliptan group's hemoglobin A1c decrease of 0.34 was not significant (range −0.99–+0.30; *P* = 0.29). There were no differences in the rate of severe hypoglycemia. Therefore, as an adjunct to metformin, empaglifozin represents another valuable treatment of type 2 diabetes, especially in pediatric patients who are often fearful of injections.

Data regarding safety and efficacy of SGLT2 inhibitor use in pediatric patients is gradually beginning to accrue, frequently from case reports and series. A twelve-year old with Prader-Willi Syndrome and insulin resistance while on growth hormone could not be successfully managed with a variety of insulin regimens or metformin or liraglutide. She achieved glycemic control when empagliflozin was combined with liraglutide (96). Among seventeen obese pediatric patients aged 10–17 years with type 2 diabetes weighing a mean of 107 kg, the pharmacokinetics and pharmacodynamics of identical dose canagliflozin did not differ from adults (97). The DAPADream study utilized add-on Dapagliflozin in thirty type 1 diabetes patients (age 12–20 years) which was a double-blinded, placebo-controlled crossover study after two unannounced meals in patients on DreaMed closed loop insulin management. Glucose time in range (TIR) was significantly improved during dapagliflozin treatment with a total insulin dose reduction of 22% but no increase in glucagon or hypoglycemia (98). Beta hydroxy butyrate levels were statistically increased in dapagliflozin- treated patients but remained in the normal range.

### SGLT2 inhibitor dosing in the young

A recently published systematic review of pediatric use of dapagliflozin or empaglifozin reports three hundred and fifty-two patients with diabetes (*n* = 189), heart failure (*n* = 38), kidney disease (*n* = 9, discussed below), PK studies (*n* = 84), as well as glycogen storage disease-related illnesses and congenital neutropenia (*n* = 32) (99). The average age of these disease groups of SGLT2i-treated pediatric patients differs: diabetes patients were teens (mean age 14.7 ± 2.9, concurrent with the largest clinical trials enrolling age 10–17 years), the glycogen storage disease patients being the youngest including patients as young as 1 year (mean age 8.5 ± 5.1) and the heart failure/CKD group being between these (mean age 11.2 ± 6.1). For dapagliflozin, in diabetics the mean daily dose was 11.4 ± 3.7 mg/day (range 5–20) and lower for the younger heart failure/CKD patients, 6.9 ± 5.2 mg. The empagliflozin mean daily dose in the pediatric diabetes patients was 15.7 ± 7.4 mg (range 5–25 mg) compared to 0.44 ± 0.28 mg/kg (range 0.1–1.3 mg/kg) in the younger and smaller glycogen storage disease patients.

### Pediatric glycogen storage disease

The experience with SGLT2i in children with Glycogen storage disease (GSD) provides insights which may be useful to translate the kidney benefits of SGLT2i to younger age groups. Besides fasting hypoglycemia and hepatomegaly often detected in infancy, GSD type 1 can have nephromegaly, proteinuria and risk for FSGS as well as hypocitraturia and risk for nephrolithiasis (100). In GSD-1a, the enzyme glucose-6-phosphatase α catalytic subunit is impaired which catalyzes the last step of glucose release from glycogen stores leading to hepatic and renal glycogen accumulation and organomegaly. In GSD-1b, the translocase for glucose—6- phosphate is impaired so that glucose-6-phosphate is not delivered to the site of enzymatic glucose release. Clinically, GSD-1a and GSD-1b are similar except for the distinguishing hallmark of neutropenia, recurrent infections and inflammatory bowel disease in GSD-1b. The cause of the neutropenia and neutrophil dysfunction in GSD-1b was recently identified as excess levels of the glucose analog, 1,5-anhydroglucitol (1,5AG) which, like glucose 6-hosphate, requires movement by the same translocase for metabolism and elimination (101). Interestingly, SGLT2 inhibition with canagliflozin decreases urinary 1,5 AG reabsorption and increases its elimination (102). Accordingly, repurposing of empagliflozin in GSD-1b patients led to a marked decrease in 1,5 AG levels in blood, improvement in neutrophil dysfunction, and less hospitalization for infection (103–105). Even in GSD, an entity characterized by hypoglycemia, only 6.3% of patients treated with empagliflozin reported symptomatic or severe hypoglycemia (99). An international questionnaire to physicians regarding off-label empagliflozin treatment indicated 112 patients with GSD-1b being treated in 24 countries, with 80 patients aged < 18 years. Twenty patients (18%) developed level 3 hypoglycemia (<54 mg/dl) and 9 patients (8%) required hospitalization (105). This international questionnaire attests to the spread of off label SGLT2 inhibitor use for a rare pediatric disease as well as the acceptance of the associated risks in order to achieve a demonstrable improvement in health.

### Pediatric kidney disease

As in adults, it is likely that SGLT2 inhibitors will benefit CKD progression in pediatric patients. However, it is important to recognize that diabetic nephropathy or hypertensive nephrosclerosis are uncommon in the pediatric age group. Moreover, SGLT2 inhibitor treatment for renal hypoplasia/dysplasia and congenital abnormalities of the kidneys and urinary tract, CAKUT, which are common causes of progressive kidney damage in children, may be complicated by the fact that polyuria is a prominent feature of these disorders. Therefore, the generalizability of SGLT2i benefits to all pediatric CKD may be limited. Nonetheless, efforts are underway to foster early pediatric inclusion in glomerular disease trials (106). Moreover, the publication of EMPA-KIDNEY trial data and sub analyses in 2023–2024 has occasioned calls for prompt testing of SGLT2 inhibitors in pediatric CKD as well (107). A single-center short-term trial of empagliflozin in patients with CKD aged 12–25 years is currently recruiting patients in the US (SGLT2I-IN-KIDS (NCT06430684). Importantly, Boehringer Ingelheim will be soon initiating a multi-center trial of empagliflozin in pediatric chronic kidney disease patients.

Currently available data for SGLT2 inhibitor use in pediatric kidney disease is very limited. Liu et al. initiated treatment with dapagliflozin in nine pediatric patients with proteinuric kidney disease with mean age 10.4 years (range 6.4–13.8 years) (108). The degree of proteinuria ranged 1.23–6.21 g/m^2^ BSA at enrollment (mean 2.1). One patient had familial FSGS, five had Alport syndrome with mutations in *COL4A5* (four) and *COL4A3* (one), one had *PAX2* mutation, one *NUP150* mutation and nephrotic range proteinuria, and one a variant in *CLCN5* causing Dent Disease. The dosing of dapagliflozin utilized the threshold of 30 kg body weight: for weight above 30 kg, the dose was 10 mg daily; for body weight <30 kg, the dose was 5 mg daily. All patients received a stable dose of fosinopril. One patient was lost to follow up. All eight patients experienced a reduction in proteinuria (ending urine protein 0.55–4.28 g/m^2^ BSA, mean 1.5) at twelve weeks (*P* < 0.05). The percentage decrease in proteinuria was 22.6% (95% CI: 8.3–36.9%). Mean serum albumin increased from 35.3 ± 6.7 g/L to 37.5 ± 7.9 g/L (*P* < 0.05). GFR declined minimally from mean 109.2 ± 32 ml/min/1.73 m^2^ (range 63.3–163.6) to 103.8 ± 28.2 ml/min/1.73 m^2^ (range 60.8–150.6). One patient had an episode of asymptomatic bacteriuria and no patient discontinued dapagliflozin during treatment. No hypoglycemia was reported.

Another small case series encompassing younger adults has been reported for SGLT2 inhibition added to RAS inhibition in glomerular disease in hopes of prompting large prospective intervention trials (109). Two patients in this series were aged 23 and 25 years. Both showed a dramatic decrease in proteinuria with SGLT2 inhibition. In the former (with FSGS), albuminuria decreased from 530 mg/gm creatinine to 192 mg/gm creatinine after 3 months, though his serum creatinine increased from 1.66 mg/dl to 2.24 mg/dl (GFR decrease from 57 to 40 ml/min/1.73 m^2^). In the latter (with FSGS), albuminuria dropped from 4,900 to 805 mg/gram creatinine after 11 months with serum creatinine improving from 1.33 to 1.02 mg/dl attributed to recovery of nephrotic syndrome.

A larger, real-world series has recently been reported for dapagliflozin treatment in 22 pediatric patients ranging in age from 12.9–17.2 who had kidney disease and proteinuria (110). Patients had Alport syndrome (*n* = 7), medication resistant nephrotic syndrome/FSGS (*N* = 7), IgAN (*N* = 5), atypical HUS (*N* = 2) and CAKUT (*N* = 1). All were treated with RAS inhibition. Dapagliflozin dose ranged 5–10 mg per day with two thirds receiving 10 mg. After 8 months of treatment, eGFR decreased from 71.1 ml/min/1.73 m^2^ at baseline to 65.5 (*P* = 0.003). Urine protein to creatinine ratio, however, did not decrease during dapagliflozin treatment (uPCR 0.6 mg/mg before treatment to 0.7 mg/mg). A decrease in proteinuria was also not seen for any of the disease subgroups.

The very recently reported trial of SGLT2 inhibition in Alport Syndrome (*N* = 112) included ten pediatric patients aged 15 ± 3 years (81). They were treated with dapagliflozin 10 mg daily (*N* = 9) and 5 mg daily in the youngest patient, aged 9. Blood pressure was stable and GFR decreased minimally, remaining above 100 ml/min/1.73 m^2^. Two pediatric patients had overt proteinuria which improved with dapagliflozin, from urine albuminuria 1,426 ± 1,247 mg/g creatinine to 641 ± 190 after 4 ± 5 months of treatment.

Taken together, these retrospective, pediatric case series in predominantly glomerular disorders show considerable variation in proteinuria reduction during SGLT2 inhibitor treatment but somewhat less improvement than some similar studies in adults. The Immunology Working Group of the European Renal Association and the Spanish Group for the Study of Glomerular Diseases reported more striking results for SGLT2 inhibition in adults with glomerulonephritis (36). Among a retrospective, observational cohort of four-hundred and ninety-three adult glomerulonephritis patients already on RAS inhibition, treatment with SGLT2 inhibition resulted in reductions of proteinuria of 35%, 41%, 45%, and 48% at 3,6,9 and 12 months of treatment. Those achieving >30% proteinuria reduction had a slower decline in GFR (−3.7 vs. −5.3 ml/min/1.73 m^2^/year; *P* = 0.001). More proteinuria reduction correlated with higher BMI. Whether or not similar results to these will be seen in larger pediatric cohorts will require well designed and funded trials.

## Practical considerations for the use of SGLT2 inhibitors

With the rapid advance of favorable clinical trial data regarding the use of SGLT2 inhibitors in adults with kidney disease, there is a reasonable expectation that their benefits would extend to patients under age 18. Their use, though, brings with it certain pragmatic considerations around “best practice” at the time of prescribing.

### Selecting an SGLT2 inhibitor

There are currently five SGLT2i that are commercially available for clinical use—canagliflozin, dapagliflozin, empagliflozin, ertugliflozin, and bexagliflozin. Each has been studied in varying populations, with an emphasis on diabetic and then cardiovascular outcomes. While each agent boasts slightly different outcomes among their trials, similar effect sizes were observed. In three RCTs conducted with primary renal outcomes, all showed overwhelming efficacy with respect to improved eGFR slopes with all three trials ending early due to efficacy (28–30). Given the similar effect sizes for both cardiovascular and renal outcomes, the observed clinical benefits of SGLT2i use is likely a class effect. Within the current landscape of clinical practice, where insurers or payors often have medication formulary restrictions that have a significant impact on patients' ability to afford these agents, it is wise to select the agent with the lowest out-of-pocket cost. Pragmatically, this often results in a reliance on the electronic medical record to advise which agent falls into the highest formulary tier or for patients or office staff to communicate with insurers to assist with selection. Empaglifozin was approved in the US in 2023 for use in pediatric type 2 diabetic patients down to age 10 (94). Innovation in pediatric subspecialty medicine has historically been advanced through off label drug use, so the use of SGLT2 inhibitors in pediatric patients without diabetes is likely to follow a similar pattern. Whether pediatric approval for Type 2 diabetes will mean empaglifozin is easier to obtain for pediatric patients than other SGLT2 inhibitors remains to be seen.

### Patient counseling with SGLT2 inhibitor initiation

At the time of prescribing, counseling should focus on the potential benefits emphasized in previous sections of this class of agents. Emphasis on the observed slowing of chronic kidney disease in clinical trials done among adults can be coupled with discussion of adjunct cardiovascular and diabetic benefits in appropriate patient populations. The possible adverse events, anticipated side effects should also be discussed, along with counseling regarding practical strategies to use to mitigate those risks.

It is important to discuss the risk of volume depletion due to the diuretic effect of SGLT inhibition. This discussion should include advice on monitoring for symptoms of orthostatic hypotension, with counseling on corrective action if experienced. Counseling at the time of prescribing can encourage optimal hydration habits and perhaps reduce discontinuance of the drug. It is also sensible to provide patients with a “sick plan” for appropriate therapy interruptions. The SGLT2i inhibitor can be held during periods of illness or perioperatively. Among appropriate populations, attention must also be given to discussing medication use during periods of fasting. Extreme caution must be advised for the use of SGLT2 in patients with pre-existing polyuria, polydipsia and/or salt wasting phenotypes of kidney disease such as renal hypoplasia, dysplasia or posterior urethral valves which are common causes of pediatric chronic kidney disease.

There has been an observed increased risk of genital mycotic infections (GMI)—vulvovaginitis, and balanitis. These are more common in women and those with any personal history of mycotic genital infections, with lowest incidence observed in circumcised men (111). Within early clinical trials, the rates of GMI ranged between approximately 5% and 10%. This was more than observed among those treated with placebo and resulted in the FDA recommending issuing a safety announcement around the prescribing of these agents. In the DINAMO study using empagliflozin in diabetic patients aged 10–17 years, a single hospitalization was reported, for skin candida infection –its site not identified as genitourinary—so its relationship to glucosuria cannot be ascertained (95). Nonetheless, when prescribing SGLT2 inhibitors, focused counseling regarding personal hygiene practices has been shown to reduce the likelihood of early infectious issues (112) and bears discussion during initial counseling.

Controversy exists as to whether the risk of urinary tract infections is increased among patients treated with SGLT2i, with both single studies as well as meta-analyses being contradictory. One recent meta-analysis showed that dapagliflozin at 10 mg daily increased the risk of UTI (Odd Ratio 1.29; 95% CI: 1.15–1.46) but that dapagliflozin at 5 mg or 2.5 mg daily did not (113). An earlier, larger meta-analysis did not show a significant risk for UTI in SGLT2i- treated vs. untreated (Relative Risk 1.05; 95% CI: 0.98–1.12) (114). Because meta-analyses of trial data may be underpowered to detect adverse events and may exclude higher risk patients, analyses of large patient databases may be useful. In one such analysis of >200,000 patients in two cohorts, risk of severe UTI (hospitalization, sepsis, pyelonephritis) was not increased in patients treated with SGLT2i compared to other second line hypoglycemic agents (Hazard Ratios 0.98 and 0.72 for the two cohorts) (115). Such data is being carefully examined and marshalled in the renal and cardiac communities to provide reassurance to patients and physicians for the use of SLGT2 inhibitors and continuation after UTI so as not to deny their benefits unnecessarily (116, 117). It has been hypothesized that the bacterial growth-promoting effect of glucose in the urine caused by SGLT2 inhibition may be countered by the diuresis, acting to reduce bacterial load (118). The development of Escherichia coli septicemia in an elderly man with bladder outlet obstruction treated with dapagliflozin may be cautionary for the use of SGLT2i in pediatric patients with CKD associated with abnormalities of the urinary tract (119).

### Monitoring and anticipated changes

Guidelines vary in their recommendation for serial laboratory monitoring following prescription of an SGLT2 inhibitor. Routine laboratory monitoring of creatinine after initiation of an SGLT2i is not strictly defined. It is our practice in adults to follow a basic metabolic panel at four to eight weeks, then at the next routine follow up visit. Within those who have not yet reached adulthood, other experts have recommended also monitoring hepatic function given ongoing maturation as well as for any early signals of genitourinary infections (120). After the initiation phase, clinicians should continue routine clinical monitoring of renal function according to their clinical judgement.

**Figure 2:**
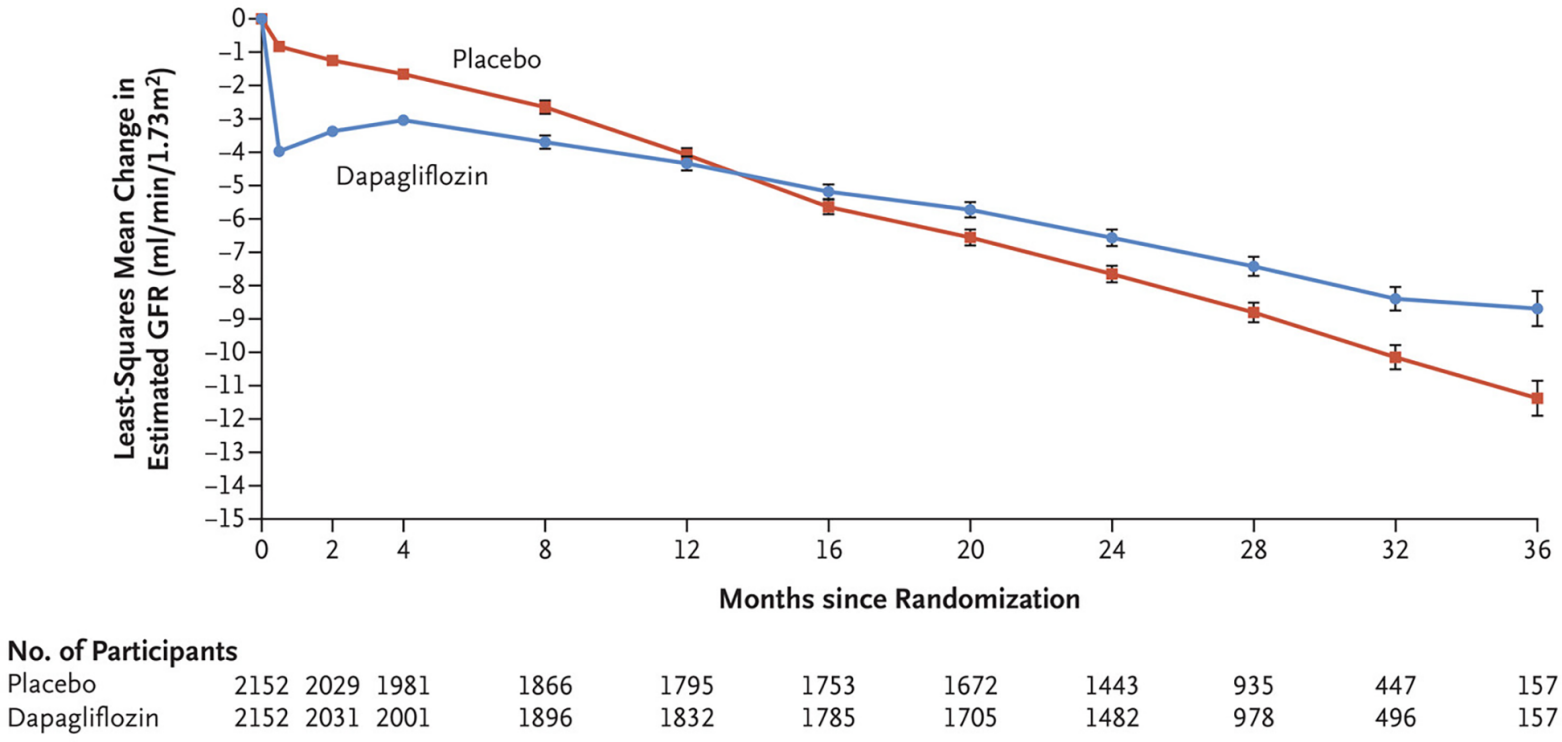
Change in estimated GFR (eGFR) from baseline in the DAPA-CKD trial. With permission from Ref. (29). The mean eGFR at baseline for the dapagliflozin group was 43.2 ml/min/1.73 m^2^ and 43 ml/min/1.73 m^2^ in the placebo group. Dapagliflozin treatment results in a prompt drop in eGFR but a slower decrease in eGFR over time, mitigating CKD progression.

It is important to acknowledge that there is an expected rise in serum creatinine after initiation of therapy. This rise is hypothesized to relate to the induced intraglomerular hemodynamic changes that come with therapy initiation as mentioned previously. Among the large clinical trials done for each agent, the median drop on eGFR after SGLT2i initiation ranged between 3 and 6 ml/min per 1.73 m^2^ (Figure 2). Like the decline in eGFR observed with initiation of ACEi/ARB, this acute decrement is balanced by favorable change eGFR slope over time. In a post-hoc analysis of the EMPA-REG Outcome trial a significant “dipping” of eGFR—defined as a decrement of ≥10 ml/min per 1.73 m^2^—was observed in 28% of trial participants during weeks 2–4 after initiation, with 41% of patients seeing an eGFR decline of 0–10 ml/min per 1.73 m^2^. These patients were older with longer duration of diabetes and less controlled hypertension. Among those with initial observed decline, some “recovery” of eGFR by week 12 was observed among all groups; further, “dippers” saw the same cardiovascular benefits as observed larger trial group (121).

In practice, our group generally accepts a serum creatinine rise of up to 25%–30%. Changes greater than that prompt a volume assessment and additional hydration counseling or concurrent medication adjustments where appropriate (i.e., diuretics). If concurrent modifiable contributors to serum creatinine rise are identified, adjustments with continued SGLT2i use often leads to reassurance with repeat labs in 2–4 weeks. If no obvious alternative cause can be identified, a medication hold with repeat laboratory studies after 2–4 weeks is reasonable. In our experience, there are a small number of patients who cannot continue. Published algorithms for surveillance of kidney function after initiation of SGLT2 inhibitor therapy offer similar guidance (122).

Consideration of the likelihood of some decrease in eGFR with SGLT2 inhibitor therapy raises the issue of the lower limit of kidney function below which SGLT2i are better avoided. Drug manufacturers and studies differ slightly in their guidance. For canagliflozin, the manufacturer discourages drug initiation in patients with eGFR less than 30 ml/min/1.73 m^2^, though continuation of drug if eGFR falls to this level during therapy is allowed. For the makers of dapagliflozin and empagliflozin, the lowest GFR levels recommended for initiation of therapy are 25 and 20 ml/min/1.73 m^2^ respectively. In adults, our practice uses these agents routinely in those with an eGFR above 25 ml/min/1.73 m^2^ and with caution in those with an eGFR between 20 and 25 ml/min/1.73 m^2^. Given the complexity of managing other burdens—physiologic, psychological, and logistical—we generally do not consider the addition of SGLT2i below an eGFR of 20- ml/min/1.73 m^2^.

## Conclusions

The discovery of the coupling of sodium and glucose absorption in the gut has been called “potentially the most important medical advance” in the 20th century as it informed oral rehydration therapy by which enteral glucose allowed sodium and water absorption and increased survival of diarrheal illness especially in resource limited environments (123–125). In the 21st century, the worldwide obesity epidemic means that diabetes, CKD, and heart disease together now contribute to mortality and functional decline more than diarrheal illness. SGLT2 inhibitors were developed to block the co-transport of sodium and glucose in the renal tubule to increase renal glucose excretion as a treatment for diabetes. In adults, SGLT2 inhibitors improve renal hemodynamics and prolong kidney function by increasing distal sodium delivery. Limited pediatric data have emerged indicating safety of SGLT2 inhibition and some reduction of proteinuria. The development of randomized, controlled trials in pediatric kidney disease are crucially needed to determine if SGLT2 kidney benefits observed in adults will translate to pediatric patients.
